# Asthma and treatment with inhaled corticosteroids: associations with hospitalisations with pneumonia

**DOI:** 10.1186/s12890-019-1025-1

**Published:** 2019-12-19

**Authors:** Emil Ekbom, Jennifer Quint, Linus Schöler, Andrei Malinovschi, Karl Franklin, Mathias Holm, Kjell Torén, Eva Lindberg, Deborah Jarvis, Christer Janson

**Affiliations:** 1 Department of Medical Sciences, Respiratory, Allergy and Sleep Research, Uppsala University, Uppsala, Sweden; 2 Population Health and Occupational Disease, National Heart and Lung Institute, Imperial College, London, UK; 3 Department of Occupational and Environmental Medicine, University of Gothenburg, Gothenburg, Sweden; 4 Department of Medical Sciences: Clinical Physiology, Uppsala University, Uppsala, Sweden; 5 Department of Surgical and Perioperative Sciences, Umeå University, Umeå, Sweden

## Abstract

**Background:**

Pneumonia is an important cause of morbidity and mortality. COPD patients using inhaled corticosteroids (ICS) have an increased risk of pneumonia, but less is known about whether ICS treatment in asthma also increases the risk of pneumonia. The aim of this analysis was to examine risk factors for hospitalisations with pneumonia in a general population sample with special emphasis on asthma and the use of ICS in asthmatics.

**Methods:**

In 1999 to 2000, 7340 subjects aged 28 to 54 years from three Swedish centres completed a brief health questionnaire. This was linked to information on hospitalisations with pneumonia from 2000 to 2010 and treatment with ICS from 2005 to 2010 held within the Swedish National Patient Register and the Swedish Prescribed Drug Register.

**Results:**

Participants with asthma (*n* = 587) were more likely to be hospitalised with pneumonia than participants without asthma (Hazard Ratio (HR 3.35 (1.97–5.02)). Other risk factors for pneumonia were smoking (HR 1.93 (1.22–3.06)), BMI < 20 kg/m2 (HR 2.74 (1.41–5.36)) or BMI > 30 kg/m2 (HR 2.54 (1.39–4.67)). Asthmatics (*n* = 586) taking continuous treatment with fluticasone propionate were at an increased risk of being hospitalized with pneumonia (incidence risk ratio (IRR) 7.92 (2.32–27.0) compared to asthmatics that had not used fluticasone propionate, whereas no significant association was found with the use of budesonide (IRR 1.23 (0.36–4.20)).

**Conclusion:**

Having asthma is associated with a three times higher risk of being hospitalised for pneumonia. This analysis also indicates that there are intraclass differences between ICS compounds with respect to pneumonia risk, with an increased risk of pneumonia related to fluticasone propionate.

## Background

Pneumonia is an important cause of morbidity and mortality. In a recent study from the United States the annual incidence was 24.8 patients hospitalized with community acquired pneumonia per 10,000 adults [1], whereas in Iceland, the incidence of community acquired pneumonia requiring hospitalisations was 20.6 cases per 10,000 adults/year [2]. Pneumonia is a common complication in patients with COPD. In a study of COPD patients from Swedish primary care over 40% had had a least one episode of pneumonia during the 8 year observation period [3]. Pneumonia is also relatively common in asthma. In a recent observational study we found that the cumulative incidence of pneumonia was 16% during the seven year study period [4]. Other studies have found that subjects with asthma have a two to three times higher risk of pneumonia than subjects without asthma [5, 6]. Smoking, gastroesophageal reflux and heart disease have also been identified as risk factors for pneumonia [5, 6].

Treatment with inhaled corticosteroids is used in both asthma and COPD. In asthma, in particular there is good evidence that ICS reduces exacerbations, and so they are included in every major guideline [7]. In COPD, treatment with inhaled corticosteroids (ICS) increases the risk of pneumonia [8, 9]. There is also an indication that this association may depend on the type of ICS prescribed, with several studies finding a higher risk for fluticasone propionate than budesonide [10–12]. In a systemic review Kew and Seniukovich found high quality evidence that fluticasone increased pneumonia events by 18 more per 1000 treated over 18 months, but there was less evidence for budesonide, with six more events per 1000 treated over nine months [13]. In asthma, however, the association between the use of ICS and pneumonia is less clear. O’Byrne and co-workers found no association between the use of budesonide and pneumonia, [14] whereas McKeever et al found a dose response relationship between the use of ICS and risk of pneumonia in asthmatics from primary care [15]. In that study an increased risk of pneumonia was found in patients treated with fluticasone propionate but not in those treated with budesonide. Recently Qian et al also found a dose related association between ICS use and pneumonia in asthma, but in that study this was found for both fluticasone propionate and budesonide [16].

The aim of this investigation was to examine risk factors for hospitalisations with pneumonia in the general population and to investigate risk factors for pneumonia in subjects with asthma with special emphasis on the use of ICS.

## Method

### Population and methods

The European Community Respiratory Health Survey (ECRHS) stage I took place in 1990–1994. In the study, males and females aged 20–44 years were randomly selected from the population register in participating centres [17]. A postal questionnaire was sent to 3000–4000 subjects at each centre.

Respiratory Health In Northern Europe (RHINE) II is a follow-up study of participants from ECRHS stage 1 from seven Northern European centres [18]. RHINE II consisted of a postal questionnaire sent in 1999–2001. The questionnaire was sent out to all participants of ECRHS stage 1 (age 28–54 years). The present analysis is based on data from the three Swedish centres: Gothenburg, Uppsala and Umea, where 7340 (79.3%) of the 9248 in the original sample participated. Written informed consent was obtained from each participant and the study was approved by the Regional Ethical Review Board in Uppsala, Sweden (1998/495).

**Fig. 1:**
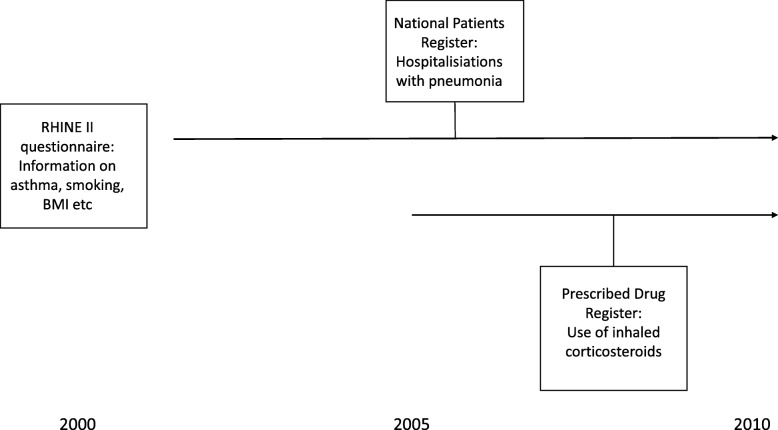
Design of the investigation

Data on inpatient treatment for pneumonia (ICD 10-Code J10-J18), including number of such hospitalisations was collected from the National Patient Register for the time period starting 1 January 2000 until 31st of December 2010 (Fig. 1). Data on date of death for the same time period was collected from the cause of death register.

Drug prescription data for hospital and primary care were collected from the Swedish Prescribed Drug Register from the time that the registry started 1st of July 2005 to the end of the study period: 31st of December 2010 (Fig. 1).

### Definition of analysed variables

A hospitalisation with pneumonia was defined as a hospitalisation that included the ICD10 code J10–18 as one of the diagnostic codes.

Current asthma was defined as answering yes to the question:” Have you had an asthma attack during the last year?” and/or” Have you taken medication for asthma during the last year?” [19]. The number of asthma related symptoms (Yes/No) was assessed where the symptoms included were wheezing, wheezing in combination with breathlessness, wheezing when not having a cold, waking up with tightness in the chest and waking up with attacks of shortness of breath. The recall period for all symptoms was 12 months.

Nasal allergy was defined as answering yes to the question “Do you have any nasal allergies including hay fever?” [19] Nocturnal gastroesophageal reflux (nGER) was defined as reported heartburn or belching at least one night per week [20]. Habitual snoring was defined as loud and disturbing snoring at least 3 nights a week [21]. Hypertension was defined as answering yes to the question “Do you have hypertension?” and heart disease was defined in a similar way. Diabetes was defined as answering yes to the question: “Has a doctor told you that you have diabetes?”

Body mass index (BMI) was calculated from the self-reported height and weight and the participants grouped into four groups, < 20, 20–25, > 25–30 and > 30 kg/m2. Smoking history was captured through the questionnaire and the participants were categorised into never-smokers, ex-smokers and current smokers. Passive smoking was defined as being exposed to tobacco smoke at home every day and not being a current smoker [21].

Use of inhaled corticosteroids (ATC code R03BA and R03AK) was captured from the Prescribed Drug Registry. The number of years that the participants had collected at least one prescription was calculated where the maximum number of years was six. In a similar way information on the use of budesonide (ATC code R03BA02 and R03AK07) and fluticasone propionate (ATC code R03BA05, R03AK06 and R03AK11).

### Statistical analysis

All analyses were performed using Stata version 14 (StataCorp, Texas, USA). Chi-2 test and unpaired t-test were used in bivariate analyses. Factors associated with the time to the first hospitalisation with pneumonia was analysed using Kaplan Meier and Cox proportional hazards models. All preselected variables were included in the model [22]. Participants were censored either at first hospitalisation with pneumonia, death or at 31st of December 2010. The proportional hazard assumption was tested for all the independent variables in the models and no violation was detected (*p* > 0.1), except for nasal allergy (*p* = 0.04).

The association between the yearly pneumonia event rate and use of ICS in participants with asthma was analysed with Poisson regression, with events as the dependent variable and number of years in the study as the offset variable. The independent variables were duration of the ICS treatment as a categorical variable, age, sex, BMI-groups, smoking history, centre and the number of asthma symptoms.

## Results

Of the 7284 subjects included in the study 119 (1.6%) had been hospitalized with pneumonia during the study period. Participants with pneumonia were more often women, older, more likely to have a BMI < 20 or > 30 kg/m2, more often smokers and had a higher prevalence of asthma and heart disease than those that had not been hospitalised (Table 1).

**Table 1:** Characteristics of participants that had not or had been hospitalised for pneumonia during the period 2000–2010 (% and mean ± SD)

	No hospitalisation (*n* = 7168)	At least one hospitalisation (*n* = 119)	*p*-value
Women	3738 (52.2)	73 (61.3)	0.047
Age	40.5 ± 7.3	43.2 ± 7.6	< 0.001
BMI			< 0.001
< 20	387 (5.4)	18 (15.4)	
20–25	3681 (51.8)	41 (35.0)	
25–30	2450 (34.5)	41 (35.0)	
> 30	582 (8.2)	17 (14.5)	
Smoking			< 0.001
Never	3620 (51.8)	48 (40.7)	
Ex	1828 (26.1)	20 (17.0)	
Current	1544 (22.1)	50 (42.4)	
Passive smoking^a^	178 (3.3)	3 (4.5)	0.58
Asthma	561 (7.8)	26 (21.8)	< 0.001
Nasal allergy	1728 (24.5)	34 (29.7)	0.22
Hypertension	459 (6.5)	11 (9.3)	0.22
Heart disease	73 (1.0)	5 (4.2)	0.001
Diabetes	131 (1.8)	3 (2.6)	0.56
Habitual snoring	1385 (20.0)	23 (20.4)	0.93
Gastroesophageal reflux	618 (8.8)	11 (9.7)	0.72

^a^Only calculated in never and ex-smokers

**Fig. 2:**
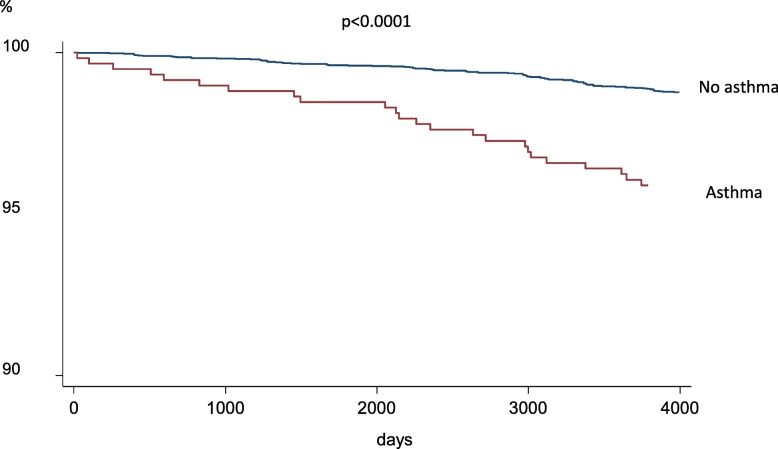
Time to first hospitalisation with pneumonia in subjects with and without asthma

Having asthma at baseline(*n* = 587) was significantly associated with a higher risk of being hospitalised with pneumonia independent of other risk factors (Fig. 2, Table 2). Apart from asthma, being hospitalised with pneumonia was independently associated with higher age, having a BMI < 20, a BMI > 30 and being a current smoker (Table 2).

**Table 2:** Association between factors measured at baseline and time to first hospitalisation with pneumonia (Hazard risk ratio (HRR) (95% confidence interval))

	Crude HRR (95% CI)	Adjusted HRR^a^ (95% CI)
Women	1.16 (0.83–1.60)	1.34 (0.88–2.05)
Age per 10 years	1.52 (1.22–1.89)	1.29 (0.97–1.73)
BMI		
< 20	3.80 (2.16–6.67)	2.74 (1.41–5.36)
20–25	1	1
25–30	1.49 (0.97–2.30)	1.53 (0.95–2.48)
> 30	2.55 (1.46–4.45)	2.54 (1.39–4.67)
Smoking		
Never	1	1
Ex	0.73 (0.43–1.24)	0.83 (0.48–1.45)
Current	1.95 (1.30–2.95)	1.93 (1.22–3.06)
Passive smoking^a^	1.50 (0.47–4.80)	1.27 (0.31–5.30)
Asthma	3.23 (2.07–5.02)	3.35 (1.97–5.70)
Nasal allergy	1.17 (0.79–1.75)	0.88 (0.54–1.42)
Hypertension	1.53 (0.92–2.86)	1.14 (0.56–2.33)
Heart disease	3.16 (1.25–7.97)	1.48 (0.46–4.79)
Diabetes	1.57 (0.50–4.94)	0.42 (0.06–3.07)
Habitual snoring	1.15 (0.72–1.83)	0.80 (0.47–1.37)
Gastroesophageal reflux	1.26 (0.67–2.35)	0.96 (0.49–1.90)

^a^Adjusted for all the variables in the table and centre

*Participants with asthma.*

During the 6-year period there were 26 hospitalisations with pneumonia among the participants with asthma and 19 patients had at least one hospitalisation with pneumonia. Those with at least one hospitalisation with pneumonia were more often women, had more often a BMI < 20 and were more likely to have used fluticasone propionate every year than those without a hospitalisation (Table 3). No significant difference was found between participants that had used fluticasone propionate and budesonide every year regarding age, sex, smoking history or number of symptoms (all *p*-values > 0.50).

**Table 3:** Characteristics of participants with asthma that had not or had been hospitalised for pneumonia during the period 2005–2010 (n (%) and mean ± SD)

	No hospitalisation (*n* = 567)	At least one hospitalisation (*n* = 19)	P-value
Women	319 (56)	16 (84)	0.02
Age	40 ± 7	40 ± 0.75	
BMI			0.007
< 20	26 (5)	4 (21)	
20–25	268 (48)	6 (32)	
25–30	196 (35)	5 (26)	
> 30	70 (12)	4 (21)	
Smoking			0.34
Never	298 (54)	12 (63)	
Ex	140 (25)	2 (11)	
Current	117 (21)	5 (26)	
Number of Symptoms	2.7 ± 1.5	3.4 ± 1.3	0.08
ICS (years)			0.02
0	238 (42)	6 (32)	
1–5	224 (39)	4 (21)	
6	106 (19)	9 (47)	
Budesonide (years)			0.52
0	253 (44.)	8 (42)	
1–5	245 (43)	7 (37)	
6	70 (12)	4 (21)	
Fluticasone (years)			< 0.0001
0	345 (61)	7 (37)	
1–5	199 (35)	7 (37)	
6	24 (5)	5 (26)	

Having been hospitalised with pneumonia was independently associated with being female, having a BMI < 20 and having many symptoms. There was a trend between having used ICS in all of the six years during the observation period and being hospitalised with pneumonia (IRR (95% CI) 2.48 (0.92–6.68)) (Table 4).

**Table 4:** Independent risk factors for being hospitalised with pneumonia during July 2005 to December 2010 in participant with asthma (incidence risk ratio (IRR) (95% confidence interval))

	IRR^a^ (95% CI)
Women	5.66 (1.26–25.3)
Age per 10 years	1.13 (0.60–2.14)
BMI	
< 20	7.80 (2.31–26.3)
20–25	1
25–30	1.83 (0.53–6.27)
> 30	2.27 (0.65–7.90)
Smoking	
Never	1
Ex	0.42 (0.09–1.94)
Current	1.73 (0.69–4.35)
Number of Symptoms	1.41 (1.01–1.95)
ICS (years)	
0	1
1–5	0.36 (0.09–1.40)
6	2.48 (0.92–6.68)

^a^Adjusted for all the variables in the table and centre

**Fig. 3:**
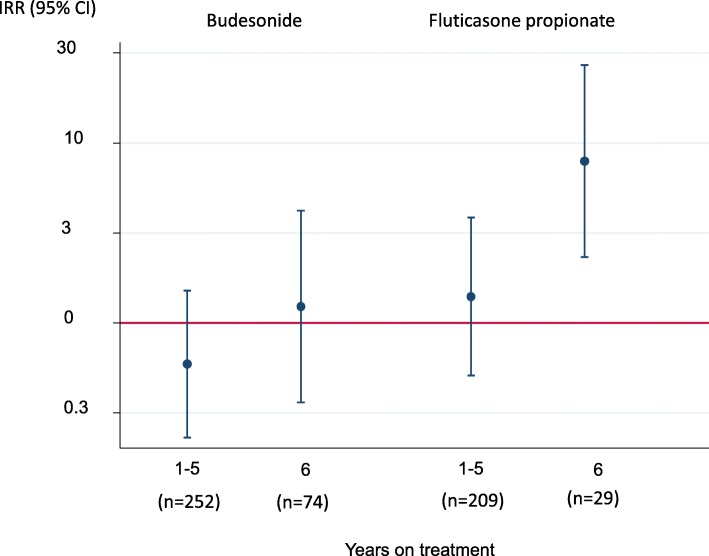
Independent association between type of inhaled corticosteroid and hospitalisations with pneumonia (incidence risk ratio* (IRR) (95% confidence interval)). Asthmatics not using respectively medication was the reference group. *adjusted for sex, age, BMI, smoking history, symptoms and centre)

There was an independent association between having used fluticasone propionate (IRR (95% CI) 7.92 (2.32–27.0)) during the whole observation period and hospitalisations with pneumonia, whereas no such association was found for budesonide (IRR (95% CI) 1.23 (0.36–4.20)) (Fig. 3).

## Discussion

The main finding of this investigation is that having asthma is associated with a three times higher risk of being hospitalised with pneumonia. Among subjects with asthma, the risk of being hospitalised with pneumonia was higher in those that were underweight and those with more symptoms. Among asthmatics, an increased risk of pneumonia was found in those treated with fluticasone propionate during the whole observation period whereas treatment with budesonide was not associated with being hospitalised with pneumonia.

The increased risk of pneumonia in subjects with asthma is in accordance with other studies [5, 6, 23]. There is also data suggesting that subjects with asthma may be more prone to get non-respiratory infections [23]. The reason for the increased risk of infections in asthma is not totally understood but an impaired immune response against bacterial infection might be part of the explanation [24]. Despite the increased risk, it should be emphasized that only 4% of participants with asthma were hospitalized with pneumonia during this 10 year period and that the risk is much lower than for instance patients with COPD [3].

Like many other studies, we found that being a current smoker is associated with an increased risk of pneumonia [5, 6, 25, 26]. The reason for this is that smoking causes both morphological changes in the airway and inflammatory reactions including inhibition of innate and adaptive response to infections [27]. Passive smoking has also been associated with pneumonia in some studies [28]. We, however, found no association between passive smoking and pneumonia in non-smokers in the present study. The most likely explanation for this is the very low prevalence of passive smoking in this population [29].

In the present study, participants that were underweight and obese had an increased risk of being hospitalised with pneumonia. This is in accordance with some other studies [5, 26, 30]. The reason is probably that both being underweight and obesity may affect the immune system [30, 31]. Pneumonia has also been associated with other obesity related disorders such as obstructive sleep apnea [32] and nGER [33], but in the present study we found no association between habitual snoring or nGER and being hospitalised with pneumonia. Other studies have shown a higher risk of pneumonia in subjects with diabetes and cardiovascular disease [34]. This was not the case in the multiple variable analysis in our study and this is probably related to the relative young age of the population.

In the present study, we found that participants with asthma that had been using ICS every year had an almost three times higher risk of being hospitalised with pneumonia during the study period. Even though this association was of only of borderline significance the result is in accordance with what has been seen in other studies [15, 16, 35]. The risk was even higher in asthmatics that had been using fluticasone propionate each year while no increased risk was found in those using budesonide. This result is in accordance with what has been seen in COPD [10, 11, 36]. This result is also in accordance with that of O’Byrne et al were no increased risk was found between treatment of budesonide and pneumonia in asthma [14] and also to the results from McKeever and co-workers where the risk of pneumonia was higher with fluticasone propionate than budesonide [15]. The results are, however, not in accordance with those of Qian et al where the adjusted risk for pneumonia was higher for budesonide than fluticasone propionate [16]. No direct comparison of pneumonia risk between budesonide and fluticasone propionate was done in any of the two studies above. A difference between the present study and the studies by McKeever et al. an Qian et al. is that they studied all kinds of pneumonia event while the present study only studied hospitalisations with pneumonia. If there is an intraclass difference between budesonide and fluticasone propionate in relation to the risk of pneumonia in asthma, this is probably explained by the difference in drug pharmacokinetics. Fluticasone propionate is less water-soluble and therefore more slowly dissolved from the airway luminal fluid than budesonide. This results in a more protracted immunosuppressive effect locally with fluticasone propionate than budesonide [37].

The strength of this investigation is that the data are from a longitudinal study with detailed information on a large number of potential risk factors at baseline. It is also a strength that we were able to combine that data with data from the national registries. We also believe that using hospitalisation with pneumonia is a strength as this makes the risk of misdiagnosis smaller as radiography is a standard procedure in investigation of pneumonia in inpatient care in Sweden [38]. The weakness is that the number of events was low which especially causes a problem when analysing the relation between ICS and pneumonia in asthma. We were therefore only able to identify factors with a high magnitude of risk. Another potential weakness is that all data, except the hospitalisations and medication data, was based on self-reported data which carries a risk of misclassification. Furthermore, apart from number of symptoms, which was only collected at one time point; we have not adjusted our analyses for asthma severity or control. Other residual confounders include treatment with oral corticosteroids and compliance to the collected ICS prescriptions.

## Conclusion

We conclude that having asthma is associated with a three times higher risk of being hospitalised for pneumonia and that the risk of being hospitalised with pneumonia was increased in asthmatics that were underweight. This analysis also suggests an intraclass differences between ICS compounds when it comes to the risk of pneumonia, with an increased risk of pneumonia related to fluticasone propionate than for budesonide. The result concerning this intraclass difference was, however, based on a low number of events and should be confirmed in large clinical trials.

## Data Availability

The dataset is still subject to further analyses, but will continue to be held and managed by the Department of Medical Sciences, Uppsala University, Uppsala, Sweden. Relevant anonymised data are available on reasonable request from the authors. The questionnaire can be downloaded from the study website: www.rhine.nu

## Abbreviations

BMI: Body mass index
COPD: Chronic obstructive pulmonary disease
ECRHS: European Community Respiratory Health Survey
HR: Hazard ratio
ICS: Inhaled corticosteroids
IRR: Incidence risk ratio
nGER: Nocturnal gastroesophageal reflux
RHINE: Respiratory Health In Northern Europe
